# Antiviral Activity of Resveratrol against Human and Animal Viruses

**DOI:** 10.1155/2015/184241

**Published:** 2015-11-29

**Authors:** Yusuf Abba, Hasliza Hassim, Hazilawati Hamzah, Mohamed Mustapha Noordin

**Affiliations:** ^1^Department of Veterinary Pathology and Microbiology, Faculty of Veterinary Medicine, Universiti Putra Malaysia, 43400 Serdang, Selangor, Malaysia; ^2^Department of Veterinary Pathology, Faculty of Veterinary Medicine, University of Maiduguri, PMB 1069, Maiduguri, Borno State, Nigeria; ^3^Department of Veterinary Preclinical Sciences, Faculty of Veterinary Medicine, Universiti Putra Malaysia, 43400 Serdang, Selangor, Malaysia

## Abstract

Resveratrol is a potent polyphenolic compound that is being extensively studied in the amelioration of viral infections both* in vitro* and* in vivo*. Its antioxidant effect is mainly elicited through inhibition of important gene pathways like the NF-*κβ* pathway, while its antiviral effects are associated with inhibitions of viral replication, protein synthesis, gene expression, and nucleic acid synthesis. Although the beneficial roles of resveratrol in several viral diseases have been well documented, a few adverse effects have been reported as well. This review highlights the antiviral mechanisms of resveratrol in human and animal viral infections and how some of these effects are associated with the antioxidant properties of the compound.

## 1. Introduction

Resveratrol (RSV) is a naturally occurring polyphenol stilbene found mostly in fermented grapes, mulberry, red wine, and peanuts. It is available in the trans- and cis-isomer forms; however, the cis-resveratrol isomer is unstable and easily transformed into the trans-form when reacted to light. It is insoluble in water but soluble in polar solvents such as ethanol and dimethyl sulfoxide. Resveratrol scavenges for superoxide and hydroxyl* in vitro*, as well as lipid hydroperoxyl radicals [1]. Previous studies have shown resveratrol to enhance longevity, regulate lipid levels, and act as a prophylactic compound against cancers and related viral infections [2]. It also attenuates superoxide generation in the mitochondria and inhibits mitochondrial dysfunction induced by arachidonic acid [2, 3]. The antiviral mechanisms and effects of RSV have been widely studied in a number of viruses which include influenza virus, hepatitis C virus [4], respiratory syncytial virus [5–9], varicella zoster virus [10], Epstein-Barr virus [11, 12], herpes simplex virus [13–16], human immunodeficiency virus [17, 18], African swine fever virus, enterovirus, human metapneumonia virus, and duck enteritis virus and in multiple sclerosis, whose animal models can be induced by viral infection. In almost all of these studies, RSV showed remarkable recession of the viral infection with the exception of multiple sclerosis and hepatitis C, where disease progression was worsened following administration of RSV [4, 19].

## 2. Structure, Bioavailability, and Function

**Figure 1:**
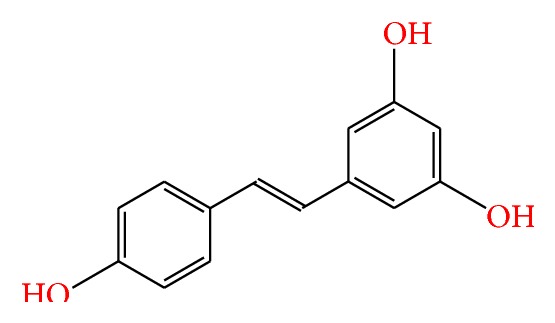
Chemical structure of resveratrol.

Resveratrol (RV), or 3,5,4′-trihydroxy-trans-stilbene, is a natural bioflavonoid compound found in plants and fruits. Its chemical structure is made up of two phenolic rings which are bonded by a double styrene bond, thus forming the 3,5,4′-trihydroxystilbene with a molecular weight of 228.25 g/mol (Figure 1). Apart from its natural isomers, cis- and trans-forms, several synthetic and natural analogs of RSV exist, which exhibit similar or slightly varying pharmacological properties to RSV [20].

Resveratrol has poor water solubility and poor oral bioavailability and rapidly metabolized in the system. Its poor bioavailability is attributed to its rapid metabolism in the liver into glucuronides and sulfates [21]. Even though the amount of oral dosing of RSV did not significantly affect its bioavailability in plasma, the type of food consumed and intraindividual differences in metabolism were shown to significantly affect its bioavailability [20, 22]. In a related study, the plasma bioavailability of RSV 30 minutes after oral consumption of red wine was only in trace amounts, while moments later RSV glucuronides were systemically abundant for a prolonged time [23]. Recently, research has been focused on developing structured nanoparticles that will enhance the bioavailability of RSV and prolong its release* in vivo*. Solid lipid nanoparticles (SLNs) and nanostructured lipid carriers (NLCs) loaded with RSV were shown to have an entrapment efficiency of 70% and stability lasting for over 2 months.* In vitro* simulation studies showed a slow sustained release of RSV at both stomach and intestinal pH levels [24]. Similarly, the use of zein-based nanoparticles was reported to enhance the* in vivo* delivery of RSV in mouse model of endotoxic shock [25].

Resveratrol has been shown to induce apoptosis by upregulating and downregulating numerous genes that are important in cellular function including but not limited to TRAIL-R2/DR5, TRAIL-R2/DR4, p53, Bim, Noxa, PUMA, Bak, Bax, Mcl-1, survivin, Bcl-XL, and Bcl-2. RSV has been shown to inhibit cellular growth at G1 and G1/S phases as well as be an anti-inflammatory mediator by inhibition of nuclear factor-kappa *β* (NF-*κβ*) activity, procyclooxygenase-2 activity, and prostaglandin production. Furthermore, its actions in delaying the onset of cardiovascular diseases and cancer progression as well as antiviral effects have been widely studied as well [2, 20].

## 3. Mechanism of Antioxidant Action

Resveratrol (RSV) scavenges for O_2_^−^ and OH^−^ *in vitro* as well as lipid hydroperoxyl free radicals. One of its most important antioxidant effects is elicited through the inhibition of reactive oxygen species production and glutathione depletion. It also attenuates superoxide generation in the mitochondria and inhibits mitochondrial dysfunction induced by arachidonic acid. These actions have thus resulted in restoration of mitochondrial membrane through mediation of the AMPK-mediated inhibitory phosphorylation of GsK3*β* downstream of poly(ADP-ribose) polymerase-LK*β*1 pathway [26, 27]. RSV also increased the levels of catalase, superoxide dismutase, and heme-oxygenase-1 in cultured epithelial cells exposed to H_2_O_2_. This shows its remarkable ability in mediating the major antioxidant enzymes involved in the breakdown of H_2_O_2_ [28]. Similarly, RSV has been shown to have an 89.1% inhibition of lipid peroxidation of linolenic acid emulsion and has strong scavenging activities against 2,2-diphenyl-1-picrylhydrazyl, 2,2-azinobis-(3-ethylbenzothiazoline-6-sulfonic acid), N,N-dimethyl-p-phenylenediamine, O_2_^−^, and H_2_O_2_. It also has a high reducing power and Fe^2+^ chelating activity [3]. However, a recent study showed that the antioxidant effect of RSV is largely dependent on its dose as higher doses were shown to increase ROS production resulting in mitochondrial-dependent death in endothelial cells [29]. It could be thus said that RSV acts as both an antioxidant and a prooxidant depending on the dose administered.

## 4. Prophylactic and Therapeutic Uses in Virus Associated Conditions

The antiviral effects of RSV have been demonstrated in a number of pathogenic human and animal viruses. However, in most of these reports, inhibition of virus proliferation was not directly associated with its antioxidant activity but its ability to inhibit viral protein production and gene expression at various levels [41, 42].

### 4.1. Influenza Virus

In influenza virus infection, RSV was shown to actively block nuclear-cytoplasmic translocation of viral ribonucleoproteins in MDCK cells, thus decreasing the expression of late viral proteins related to inhibition of protein kinase C associated pathways. This activity was also found to be unassociated with glutathione-mediated antioxidant activity of the compound [30]. The various mechanisms of viral inhibition exerted by resveratrol on certain viruses are summarized in Table 1.

### 4.2. Epstein-Barr Virus

In Epstein-Barr virus (EBV) infection, RSV showed an enhanced inhibitory effect on EBV early antigen induction using Raji cells. It was also shown to reduce papilloma production in mouse by 60% after 20 weeks of inoculation [31]. In another study, RSV was shown to dose-dependently inhibit EBV lytic cycle by inhibition of transcription genes and proteins, Rta, Zta, and diffused early antigen (EA-D), as well as inhibiting the activity of EBV immediate-early protein: BRLF1 and BZLF1 promoters. This effect was seen to reduce virion production [12]. Similarly, another* in vitro* study confirmed the previous finding that RSV does inhibit lytic gene expression and viral particle production in a dose-dependent manner. Here its main antiviral mechanism was associated with inhibition of protein synthesis, reduction in ROS production, and inhibition of transcription factors NF-*κβ* and AP1 [32]. Since EBV is one of the most renowned oncogenic viruses, it is pertinent to study the role of EBV in cellular transformation and cancer progression. RSV was thus shown to prevent transformation of EBV in human B-cells through downregulation of antiapoptotic proteins: Mc1 and survivin. This was also linked to suppression of EBV induced signaling of NF-*κβ* and STAT-3, as well as miR-155 and miR-34a in EBV infected cells [11].

### 4.3. Herpes Simplex Virus

RSV was shown to inhibit the replication of herpes simplex virus-1 and herpes simplex virus-2 (HSV-1 and HSV-2) in a dose-dependent and reversible way. In this study, the authors observed a reduction in virus yield as a result of inhibition of an early event in the replication cycle: decreased production of early viral protein ICP-4. RSV also delayed interphase stage of the cell cycle and prevented virus reactivation in neuron cells that were latently infected [15]. In another study by the author using nude mice, topical application of 12.5% and 15% resveratrol ointment suppressed the development of cutaneous lesions in abraded skin infected with HSV-1 [16]. Similarly, application of 19% RSV cream on the vagina of mouse infected with HSV-2 and HSV-1 completely prevented the development of vaginal lesions, while the mortality rate was 3% as compared to the placebo group where mortality rate was 37% [14]. These remarkable effects of RSV on HSV-1 and HSV-2 infections were reported to be due to the promotion of a rapid and sustained release of ROS, which resulted in the inhibition of NF-*κβ* and extracellular signal-regulated kinases/mitogen-activated protein kinases (Erk/MAPK), as well as a blockade in the expression of immediate-early, early, and late HSV genes and viral DNA synthesis [13, 33].

### 4.4. Respiratory Syncytial Virus

Respiratory syncytial virus (RPSV) infection is one of the most important viral diseases of the respiratory system affecting humans and it has no specific treatment. Administration of RSV in mice infected with RPSV reduced the accompanying inflammation and levels of interferon-gamma (IFN-*γ*). The mechanism here was attributed to control of toll-like receptor 3 expression, inhibition of toll/IL-1R domain-containing adaptor inducing IFN (TRIF) signaling, and induction of muscarinic 2 receptor (M2R) [9]. In an* in vitro* study, RSV treatment in epithelial cells inoculated with RPSV resulted in decreased production of interleukin- (IL-) 6 and a partial reduction in viral replication. There was also an inhibition of viral induced toll-like receptor domain and TANK binding kinase 1 (TBK1) protein expression [7]. RSV treatment of mice infected with RPSV was shown to increase sterile-*α*- and armadillo motif-containing protein (SARM) expression and decrease matrix metalloproteinase 12 (MMP-12) and TIR-domain-containing adapter-inducing interferon-*β* (TRIF) expression; these in turn decreased IFN-*γ* expression and airway inflammation and hyperresponsiveness (AHR) [6, 34]. In a related study, RPSV infected mice treated with RSV also showed decreased levels of inflammatory cells and AHR. However, while RSV was able to drastically reduce the levels of nerve growth factor (NGF) after 21 days of infection, the level of brain derived neurotrophic factor (BDNF) was not significantly affected in both the treated and untreated groups [9]. Combination of RSV and baicalin (a flavonoid found in numerous species of* Scutellaria*) joint enema was shown to increase the levels of tumor necrosis factor-alpha (TNF-*α*), IFN-*γ*, and IL-2 in mice infected with RPSV, which is believed to be among its antiviral mechanisms [5].

### 4.5. Human Immunodeficiency Virus

In the treatment of HIV-1, a combination of RSV and decitabine (a nucleoside metabolic disorder used in the treatment of myelodysplastic syndromes) was found to be more potent than RSV alone as an anti-HIV-1 drug. However, the research also reported 15 other derivatives of RSV that were more potent as an anti-HIV-1 drug [17]. Inhibition of replication of the HIV molecular clone NL4-3 containing the mutant M184V reverse transcriptase (RT) by RSV (5 *μ*M) was reported to be associated with inhibition DNA synthesis during the reverse transcription step of the HIV life cycle. This fact was proven when administration of RSV to NL4-3 clones without the mutant M184V RT failed to inhibit viral DNA synthesis [18].

### 4.6. Varicella Zoster and Enterovirus 71

Other viruses that were inhibited by RSV include varicella zoster, which was dose-dependently and reversibly inhibited in MRC-5 cells when added to culture within the first 30 hours of infection. Here, RSV was shown to decrease the synthesis of intermediate early protein (IE 62) [10]. Enterovirus 71 (EV 71) was also susceptible to RSV treatment as the compound effectively inhibited the synthesis of its viral protein 1 (VP1) and phosphorylation of proinflammatory cytokines (IKK*α*, IKK*β*, IKK*γ*, IKB*α*, NF-*κβ* p50, and NF-*κβ* p65) in rhabdosarcoma cell line. Secretion of IL-6 and TNF-*α* was also inhibited in the infected cells by RSV [35].

### 4.7. Duck Enteritis Virus, Human Metapneumonia Virus, African Swine Fever Virus, Human Rhinovirus, and Cytomegalovirus

Duck viral enteritis (DVE) also known as duck herpes viral enteritis or duck plague is a highly fatal disease of ducks and ducklings caused by the duck enteritis virus (DEV), a herpesvirus [43, 44]. In DVE, viral replication was impaired by RSV via suppression of nucleic acid replication and suppression of viral capsid formation* in vitro*. Production of viral protein was also suppressed within the first 24 hours following infection [36]. RSV in combination with a bioflavonoid, quercetin, was shown to reduce cellular oxidative damage and secretion of proinflammatory mediators (IL-1*α*, IL-6, and TNF-*α*) and chemokines (CXCL10 and CCL4), through suppression of NF-*κβ* and interferon regulating factor (IRF-3), as well as viral replication in human metapneumonia (hMPV) virus infection. However, RSV did not affect viral gene transcription and protein synthesis [37]. African swine fever virus (ASFV) causes an acute hemorrhagic disease in pigs that results in up to 100% mortality. Resveratrol and oxyresveratrol (a hydroxylated analog of resveratrol) were also found to have a dose-dependent effect on African swine fever virus* in vitro*. This was achieved through inhibition of early and late viral protein synthesis, reduced viral DNA replication, and virion formation. Hence a 98–100% reduction in viral titers was observed [38]. In human rhinovirus (HRV) infection of HeLa and nasal epithelial cells, RSV was found to exhibit a high dose-dependent antiviral activity against the virus, which was achieved through reversion of HRV-induced expression of ICAM-1. In addition, reduction in the secretion of IL-6, IL-8, and RANTES was also observed [39]. In an antiviral study of resveratrol on cytomegalovirus infection of human embryonic lung fibroblast (HEL 299), RSV prevented the synthesis of viral proteins and also inhibited virus induced activation of epidermal growth factor and phosphatidylinositol-3-kinase signal transduction. Furthermore, transcription factors of NF-*κβ* and Sp1 were also inhibited. These mechanisms were observed to decrease the overall replication of the virus [40].

### 4.8. Hepatitis C Virus and Multiple Sclerosis

In a study conducted by Nakamura et al. [4], RSV was found to dose-dependently enhance viral RNA replication in hepatitis C virus infection* in vitro*. Interestingly, RSV was also reported to reverse the antiviral effects of ribavirin and interferon on HCV RNA replication and was considered nontherapeutic in the treatment of HCV infection [4]. Similarly, RSV was also found to exacerbate the clinical and histological signs of viral model of multiple sclerosis (MS), induced by Theiler's murine encephalomyelitis virus (TMEV), which belongs to the Picornaviridae [19]. Sato et al. also showed that RSV also exacerbated an autoimmune model for MS, experimental autoimmune encephalomyelitis (EAE). However, such studies are few and there are more studies highlighting the beneficial effects of RSV against viral infections, rather than its deleterious exacerbatory effects.

## 5. Conclusion

Resveratrol has shown a high antiviral potential that can be explored in both human and animal viral infections. Its main antiviral mechanisms were seen to be elicited through inhibition of viral protein synthesis, inhibition of various transcription and signaling pathways, and inhibition of viral related gene expressions. Even though there are still limitations on its bioavailability following intake, which is being widely studied, more studies should be focused on its direct use in the amelioration of viral infections in humans and companion animals.

## Figures and Tables

**Table 1:** Mechanism of viral inhibition and exacerbation induced by resveratrol on different viruses.

Virus	Mode of propagation	Mechanism of resveratrol action	Effects on viral infection	References
Influenza virus	MDCK cells	Block nuclear-cytoplasmic translocation of viral ribonucleoproteins	Decrease in the expression of late viral proteins related to inhibition of protein kinase C associated pathways	[30]

Epstein-Barr virus	(i) Raji cells (ii) Mice (iii) P3HR1 cells (iv) Burkitt's lymphoma cell (v) Human B-cells	(i) Inhibition of early antigen induction (ii) Inhibition of early genes expression of lytic proteins (iii) Inhibition of lytic gene expression and viral particle production (iv) Inhibition of protein synthesis and reduction of ROS production and transcription of factors NF-*κβ* and AP1 (v) Downregulation of antiapoptotic proteins: Mc1 and survivin (vi) Suppression of NF-*κβ*, STAT-3, miR-155, and miR-34a signaling	(i) Reduce papilloma production (ii) Inhibition of viral transcription (iii) Prevents transformation of EBV	[31] [12] [32] [11]

Herpes simplex virus	(i) Vero and MRC-5 cells (ii) Mice (iii) Mice (iv) HeLa, Vero, and H1299 cells (v) Vero cells	(i) Decreased production of early viral protein ICP4 (ii) Inhibition of interphase phase and prevention of virus reactivation (iii) Rapid and transient release of reactive oxygen species (iv) Reduction of mRNA of ICP0, ICP4, ICP8, and HSV-1 DNA polymerase (v) Reduction of mRNA of glycoprotein C and HSV late gene	(i) Reduction in viral yields (ii) Suppression of development of cutaneous lesions (iii) Prevented development of extravaginal lesions (iv) Inhibition of HSV replication through ROS generation (v) Inhibition of viral transcription and DNA synthesis	[15] [16] [14] [13] [33]

Respiratory syncytial virus	(i) Mice (ii) Lung epithelial cell (iii) Mice (iv) Mice (v) Mice	(i) Control of toll-like receptor 3 expression, inhibition of TRIF signaling, and induction of M2 receptor (ii) Inhibition of viral induced toll-like receptor domain and TANK binding kinase 1 protein expression (iii) Increased SARM and decreased TRIF expression	(i) Reduction in inflammation and levels of interferon-gamma (ii) Partial reduction in viral replication and decreased production of interleukin-6 (iii) Enhanced interferon-gamma expression and airway inflammatory response (iv) Decreased level of inflammatory cells and interferon-gamma (v) Increased TNF-*α*, IFN-*γ*, and IL-2 production	[9] [7] [6] [34] [8] [5]

Human immunodeficiency virus (HIV-1)	Primary peripheral blood lymphocytes	Inhibition of DNA synthesis in NL4-3 clone with mutant M184V RT	Inhibition of HIV-1 strain replication	[18]

Varicella zoster virus	MRC-5 cell	Reduction in synthesis of protein and mRNA levels of IE62	Decrease in viral production	[10]

Enterovirus (EV 71)	Rhabdosarcoma cell line	Inhibition of synthesis of viral protein 1 and phosphorylation of proinflammatory cytokines	Inhibition of IL-6 and IFN-*γ* in infected cells	[35]

Duck enteritis virus (DEV)	Duck embryo fibroblast	Suppression of nucleic acid replication, viral capsid formation, and viral early protein expression	Inhibition of DEV in host cells	[36]

Human metapneumonia virus (hMPV)	Alveolar type 2 cancerous cell line	Suppression of NF-*κβ* and interferon regulatory factor (IRF-3)	Inhibition of viral replication and reduction in cellular oxidative damage and proinflammatory mediators	[37]

African swine fever virus (ASFV)	Vero cell	Inhibition of early and late viral protein synthesis and virion formation	Reduced viral DNA replication resulting in 98–100% reduction in viral titers	[38]

Human rhinovirus (HRV-16)	HeLa cell and nasal epithelia (*ex vivo*)	Reversion of HRV-induced expression of ICAM-1	Exhibited high dose-dependent antiviral activity against HRV, leading to reduction in secretion of IL-6, IL-8, and RANTES	[39]

Cytomegalovirus	Human embryonic lung fibroblast (HEL 299)	(i) Prevention of production of immediate-early, early, and late viral proteins (ii) Reduced viral induced activation of epidermal growth factor receptor, phosphatidylinositol-3-kinase signaling, and NF-*κβ* and Sp1 transcription factor activation	Decreased viral replication	[40]

Hepatitis C virus	OR6 cells	(i) Dose-dependently enhanced HCV viral RNA replication (ii) Reversed antiviral effects of ribavirin and interferon	Increased viral RNA replication	[4]

Theiler's murine encephalomyelitis virus (TMEV)	Mice	Significantly exacerbated demyelination and inflammation without neuroprotection in the central nervous system	(i) Exacerbated clinical signs and histological findings in TMEV infected mice (ii) Resulted in a twofold increase in IL-17 and a twofold decrease in IFN-*γ*	[19]
